# Both Hepatic and Body Iron Stores Are Increased in Dysmetabolic Iron Overload Syndrome. A Case-Control Study

**DOI:** 10.1371/journal.pone.0128530

**Published:** 2015-06-01

**Authors:** Caroline Jézéquel, Fabrice Lainé, Bruno Laviolle, Anita Kiani, Edouard Bardou-Jacquet, Yves Deugnier

**Affiliations:** 1 CHU Rennes, Liver Unit, F-35033, Rennes, France; 2 INSERM, CIC 1414, F-35033, Rennes, France; 3 University Rennes 1, Faculty of Medicine, F- 35043, Rennes, France; 4 CHU Rennes, Department of Radiology, F-35033, Rennes, France; 5 INSERM, U991, F-35033, Rennes, France; Lady Davis Institute for Medical Research/McGill University, CANADA

## Abstract

**Background & Aims:**

Hepatic iron is increased in dysmetabolic iron overload syndrome (DIOS). Whether this reflects elevated body iron stores is still debated. The study was aimed at assessing body iron stores in DIOS patients by calculating the amount of mobilized iron (AMI).

**Methods:**

We conducted a prospective case-control study comparing AMI in 12 DIOS patients and 12 overweight normoferritinemic subjects matched on BMI and age. All participants were phlebotomized until serum ferritin dropped ≤ 50μg/L.

**Results:**

The two groups were comparable with respect to metabolic abnormalities and differed according to serum ferritin levels only. AMI was significantly (p<0.0001) higher in DIOS (2.5g±0.7) than in controls (0.8g±0.3). No side effects were related to phlebotomies.

## Introduction

The dysmetabolic iron overload syndrome (DIOS)—initially coined as insulin resistance-associated hepatic iron overload by Mendler et al [1]—corresponds to mild hepatic iron excess in the context of various features of the metabolic syndrome [2,3]. It is usually diagnosed in middle-aged males presenting with moderate hyperferritinemia (< 1500 ng/ml), normal transferrin saturation, and, in half of the cases, non-alcoholic fatty liver disease (NAFLD). In daily practice, its diagnosis is made on the basis of increased serum ferritin levels with elevated hepatic iron at magnetic resonance imaging (MRI) in the absence of the usual causes of iron overload in patients with overweight, increased blood pressure, dyslipidemia, and/or abnormal glucose metabolism. Although the reality of hepatic iron excess is well documented in DIOS by both liver biopsy [4,5] and MRI [6], whether total body iron stores are elevated remains debated. Hyperferritinemia may be related to associated subclinical inflammation and/or cell necrosis [7]. MRI has disputed sensitivity and specificity in detecting and quantifying mild hepatic iron excess, especially when associated to steatosis [6,8]. Moreover, hepatic siderosis may be related to tissue iron redistribution in the absence of increased total body iron stores.

The determination of the amount of mobilized iron (AMI) is the only means to accurately measure body iron stores. Then, we conducted a prospective case-control study comparing AMI in DIOS patients and in overweight controls without hyperferritinemia.

## Materials and Methods

Twenty-four subjects—12 with DIOS and 12 overweight controls with normal serum ferritin levels—were included in the study. All were males, older than 18 years and enrolled at the University Hospital of Rennes, France. This study was approved by the regional Ethics Committee (Comité de Protection des Personnes—Ouest V, n°11/13–802—June 7, 2011) and all subjects gave an informed written consent. Sample sizes were calculated to demonstrate a difference of 0.7 g at least between the 2 groups with alpha < 0.05 and beta < 0.05.

DIOS patients were consecutively recruited at the outpatients clinics and included in the study if the following criteria were met: serum ferritin levels between 450 and 1500 μg/L, hepatic iron concentration > 50 μmol/g at MRI and BMI > 25 Kg/m^2^ in the absence of other causes of iron overload. Controls subjects were recruited through public advertising and had to fulfil the following criteria: serum ferritin levels between 100 and 300 μg/L and BMI higher than 25 Kg/m^2^. They were matched to DIOS patients according to age and BMI. Patients and controls who had a history of blood donation, blood transfusion, iron or vitamin C supplementation or alcohol consumption > 30 g per day, any chronic inflammatory disease or increased CRPus ≥ 10 were ineligible.

MRI was performed in DIOS patients to estimate liver iron content using the method described by Gandon et al. [6] and available at http://www.radio.univ-rennes1.fr. It was not performed in controls.

All subjects were proposed the same venesection program consisting of phlebotomies of 7 ml/kg every 14 (+/-1) days until serum ferritin dropped < 50μg/L. The amount of mobilized iron (AMI) was calculated as follows and expressed as grams [9]: [Removed blood volume (L)* Hb at baseline (g/L)*0.0034]–[duration of phlebotomy treatment (days)*0.002]–[(Hb at baseline (g/L)—Hb at the end of therapy (g/L))*0.0034].

Results are expressed as median and interquartile range [IQR] for continuous variables and as proportions for categorical variables. The normality of variables was evaluated by the Shapiro-Wilk test. Statistical comparisons between the 2 groups were performed using matched t-test or chi-square test as appropriate. Correlations were performed using Spearman’s test. For all analyses p < 0.05 was considered significant. Statistical analysis was conducted on SAS 9.3 (Cary, NC, USA).

## Results

Baseline characteristics of patients and controls are shown in Table 1. The two groups were comparable with respect to the nature and frequency of metabolic abnormalities and to haemoglobin and serum transaminase levels. They differed according to serum ferritin levels and transferrin saturation only.

**Table 1:** Characteristics of DIOS patients and overweight controls with normal serum ferritin levels at baseline and after iron removal.

	Controls (n = 12)	DIOS (n = 12)	p
**At baseline**			
Age (years)	57 [49–63]	56 [50–63]	0.34
Body mass index (Kg/m^2^)	28.9 [27.1–31.2]	28.4 [26.7–31.1]	0.26
Waist circumference (cm)	105 [100–109]	98 [95–111]	0.09
Systolic blood pressure (mmHg)	139 [137–145]	129 [120–145]	0.27
Diastolic blood pressure (mmHg)	88 [86–92]	85 [75–94]	0.2
Serum ferritin (μg/L)	175 [131–241]	813 [644–894]	< 0.0001
Hepatic iron (μmol/g)	NA	85 ± 14	
C reactive protein (mg/L)	1.5 [0.9–2.9]	1.6 [0.6–3.3]	0.67
ALT (UI/L)	35 [29–44]	41 [29–46]	0.4
AST (UI/L)	23 [21–29]	29 [25–35]	0.16
Serum iron (μmol/L)	18 [16–23]	21 [17–27]	0.35
Transferrin saturation (%)	23.4 [21.6–36.3]	32.3 [26.5–42.6]	0.05
Serum transferrin (g/L)	2.9 [2.7–3.1]	2.4 [2.3–2.7]	0.03
Hemoglobin (g/dL)	15.1 [14.6–15.4]	15.5 [14.6–16.3]	0.21
γ-GT (UI/L)	36 [28–80]	38 [23–52]	0.8
Total cholesterol (mmol/L)	5.3 [4.6–5.9]	5.4 [4.8–5.6]	0.57
HDL-cholesterol (mmol/L)	1.3 [1.1–1.4]	1.4 [1.3–1.4]	0.91
LDL-cholesterol (mmol/L)	3.5 [2.9–3.9]	3.4 [2.8–3.5]	0.28
Triglycerides (mmol/L)	1.1 [0.9–1.5]	1.3 [0.9–2.1]	0.27
Fasting glucose (mmol/L)	5.4 [4.9–6.1]	5.6 [5.2–5.8]	0.53
**After phlebotomies**			
Serum ferritin (μg/L)	28 [25–36]	46 [36–53]	<0.0001
Hemoglobin (g/dL)	13.4 [12.9–13.6]	14 [13.2–15]	0.07

Variables are presented as median value and interquartile range. A p value lower than 0.05 was considered as significant.

In the whole population, only 9 subjects (5 controls and 4 DIOS patients) were obese, with BMI between 30 and 35 Kg/m2. None had severe or morbid obesity. Obese and overweight subjects were comparable with respect to haemoglobin and serum transaminases levels.

All subjects achieved low body iron stores with no significant side effects, including anaemia. However, serum ferritin remained > 50 μg/l in 3 DIOS patients (56, 72 and 110 μg/l, respectively) who prematurely stopped iron removal for personal reasons unrelated to poor haematological tolerance (haemoglobin levels at the end of treatment: 13.8, 13.1 and 15.9 g/dL respectively).

**Fig 1:**
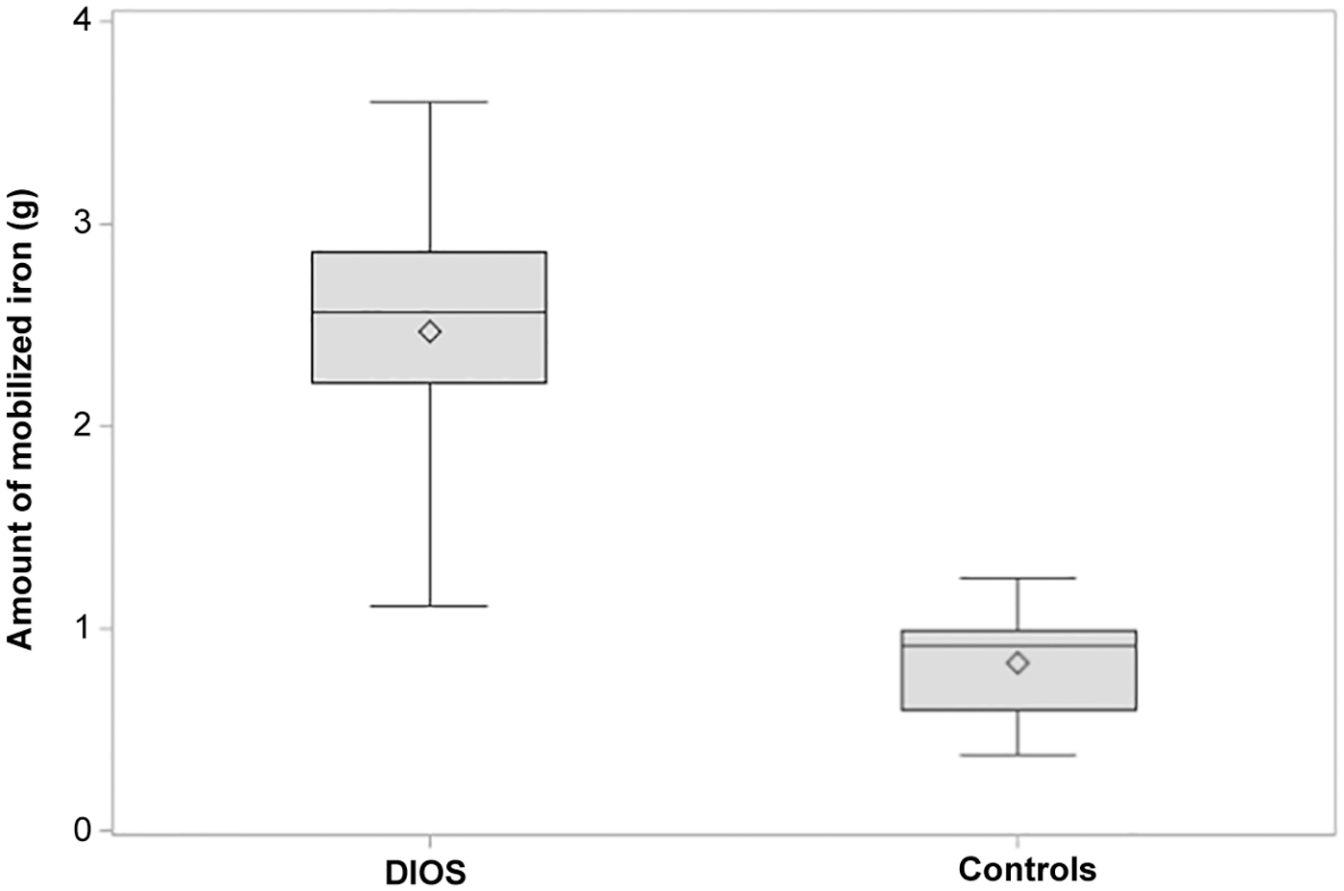
Amount of mobilized iron (g) in DIOS patients (on the left) and in overweight controls with normal serum ferritin levels (on the right). Box represents interquartile range, horizontal line inside the box indicates the median value and diamond indicates the mean value, horizontal lines above and below the box indicate maximum and minimum values.

As shown on Fig 1, AMI was significantly (p < 0.0001) higher in DIOS patients (2.5 ± 0.5 g) than in overweight controls (0.8 ± 0.3 g). In the control group, AMI did not differ in obese (0.82 ± 0.3 g) and overweight subjects (0.84 ± 0.3g).

Serum ferritin levels and AMI were positively correlated in the whole population and in controls (R2 = 0.87, p<0.0001 and R2 = 0.78, p = 0.003, respectively), but not in DIOS patients (R2 = 0.24, p = 0.5). Hepatic iron concentration and AMI were not significantly correlated in DIOS patients (R2 = 0.44, p = 0.15).

There was a significant correlation between AMI and CRPus in the DIOS group only (R2 = -0.6, p = 0.04). Otherwise, no correlation was found between AMI and CRPus in controls (R2 = 0.14, p = 0.67) and between serum ferritin and CRPus in both groups (R2 = -0.06, p = 0.85 in DIOS patients and R2 = 0.17, p = 0.6 in controls).

## Discussion

This prospective matched-controlled study demonstrates that patients diagnosed as having DIOS on the basis of hyperferritinemia and elevated hepatic iron concentration at MRI have four times higher total body iron stores than overweight controls with normal serum ferritin.

Our goal was to answer the question whether overweight patients with DIOS had elevated body iron store, which is still disputed in literature. This led us to raise a control group of overweight patients with normal serum ferritin levels. Because it was not ethical to proceed to phlebotomies in subjects with low body iron stores, overweight controls with serum ferritin levels < 100 μg/l were not included. This limits the representativeness of our controls, but in fact only a few subjects were not eligible for this reason. MRI was not performed in controls because normal ferritin levels are considered sufficient to rule out hepatic iron excess. In addition, it is well known that the sensitivity of MRI in quantifying low hepatic iron stores remains poor [6], which precluded any reliable correlation studies with other markers of iron burden. Since iron deficiency has been reported to be more frequent in obese than in non-obese subjects [10], it could be argue that our controls were iron deficient and that the difference in AMI between controls and DIOS patients was overestimated. This is unlikely because (i) iron deficiency was described in severe and morbid obese only as illustrated by the study of Yanoff et al [10] and our population consisted of subjects with BMI comprised between 25.0 and 34.1 kg/m2; (ii) haemoglobin value at baseline was 15.2 ± 0.8 g/dl in the whole population and did not differ according to BMI (15.2 ± 0.8 in obeses versus 15.1 ± 0.9 in others); and (iii) AMI did not significantly differ in the 5 obese controls (0.82 ± 0.3 g) when compared to the 7 overweight controls (0.84 ± 0.3 g). Then, we can assume that the difference in AMI between the control and DIOS groups was not related to abnormal low iron stores in controls but to increased body iron stores in DIOS patients.

Beaton et al recently published the first phase 2 clinical trial of phlebotomy in NAFLD [11]. Their patients were selected on the basis of biopsy-proven NAFLD and not of serum ferritin levels. Then, they were not strictly identical to ours, especially since NAFLD has been reported in 50% of DIOS patients only [1,4] and since a large proportion of patients with NAFLD have no increased body iron stores [5]. However, Beaton’s patients with baseline serum ferritin levels above the median (i.e. 295 μg/l) required the removal of an average of 2.34 g of iron to achieve low body iron stores (i.e. serum ferritin < 50 μg/l) compared to 1.27 g in patients with normal serum ferritin levels at baseline. This is in line with the present results and with previous studies estimating body iron stores between 0.18 and 1.35 g (mean = 0.75) in young healthy males [9]. Then, it can be concluded that our patients had a slight—when compared to genetic hemochromatosis—but indisputable increase in body iron stores and not only iron redistribution towards the liver, inflammatory hyperferritinemia [12] or false positive MRI results as previously speculated [8].

The significant correlation that we found between AMI and serum ferritin levels in the whole 24 subjects did not remain in DIOS patients when studying both groups separately. Similarly, we failed to find any correlation between AMI and hepatic iron in DIOS patients. This is likely due to the small number of patients studied as well as to the lack of precision of MRI in measuring hepatic iron excess in the low range [6]. One other explanation could be partial extrahepatic localization of iron deposition in patients with DIOS. This is strongly suggested by recent studies that demonstrated an increase of iron concentration in visceral adipose tissue in a mice model of DIOS [13,14].

Finally, our study provides an evaluation of body iron stores in subjects with moderately increased BMI and normal ferritin levels. It is of note that final serum ferritin levels being slightly but significantly higher in DIOS patients than in controls, the difference of body iron stores between the 2 groups was likely a little bigger than that we calculated. Investigations on a larger group of overweight and obese subjects would be needed to more precisely define the normal range of body iron stores according to BMI.

The present study demonstrates that total body iron stores are indisputably increased in patients with DIOS and that hyperferritinemia and elevated hepatic iron concentration at MRI do not correspond to artefacts. It reinforces the rationale for further controlled studies of venesection therapy in DIOS patients.
